# Serum lactate dehydrogenase profile as a retrospective indicator of uterine preparedness for labor: a prospective, observational study

**DOI:** 10.1186/1471-2393-13-128

**Published:** 2013-06-08

**Authors:** Jeremy L Neal, Nancy K Lowe, Elizabeth J Corwin

**Affiliations:** 1 The Ohio State University, 1585 Neil Avenue, Columbus, OH, 43210, USA; 2 Division of Women, Children, and Family Health in the College of Nursing, University of Colorado Denver, 13120 East 19th Avenue, Aurora, CO, 80045, USA; 3 Nell Hodgson Woodruff School of Nursing, Emory University, 1520 Clifton Road NE, Atlanta, GA, 30322, USA

**Keywords:** Labor, Obstetric, Lactate dehydrogenase, Nullipara, Serum, Uterus

## Abstract

**Background:**

Lactate dehydrogenase (LDH) isoenzymes are required for adenosine triphosphate production, with each of five different isoenzymes having varying proficiencies in anaerobic versus aerobic environments. With advancing pregnancy, the isoenzyme profile in uterine muscle shifts toward a more anaerobic profile, speculatively to facilitate uterine efficiency during periods of low oxygen that accompany labor contractions. Profile shifting may even occur throughout labor. Maternal serum LDH levels between 24–48 hours following delivery predominantly originate from uterine muscle, reflecting the enzymatic state of the myometrium during labor. Our purpose was to describe serum LDH isoenzymes 24–30 hours post-delivery to determine if cervical dilation rates following labor admission were associated with a particular LDH profile. We also compared differences in post-delivery LDH isoenzyme profiles between women admitted in pre-active versus established active labor.

**Methods:**

Low-risk, nulliparous women with spontaneous labor onset were sampled (n = 91). Maternal serum LDH was measured at labor admission and 24–30 hours post-vaginal delivery. Rates of cervical dilation during the first four hours after admission were also measured. Spearman’s rho coefficients were used for association testing and t tests evaluated for group and paired-sample differences.

**Results:**

More efficient dilation following admission was associated with decreased LDH_1_ (p = 0.029) and increased LDH_3_ and LDH_4_ (p = 0.017 and p = 0.017, respectively) in the post-delivery period. Women admitted in established active labor had higher relative serum levels of LDH_3_ (t = 2.373; p = 0.023) and LDH_4_ (t = 2.268; p = 0.029) and lower levels of LDH_1_ (t = 2.073; p = 0.045) and LDH_5_ (t = 2.041; p = 0.048) when compared to women admitted in pre-active labor.

Despite having similar dilatations at admission (3.4 ± 0.5 and 3.7 ± 0.6 cm, respectively), women admitted in pre-active labor had longer in-hospital labor durations (12.1 ± 4.3 vs. 5.3 ± 1.4 hours; p < 0.001) and were more likely to receive oxytocin augmentation (95.5% vs. 34.8%; p < 0.001).

**Conclusions:**

More efficient cervical dilation following labor admission is associated with a more anaerobic maternal serum LDH profile in the post-delivery period. Since LDH profile shifting may occur throughout labor, watchful patience rather than intervention in earlier labor may allow LDH shifting within the uterus to more fully manifest. This may improve uterine efficiency during labor and decrease rates of oxytocin augmentation, thereby improving birth safety.

## Background

The threshold for the active phase of labor is suggested to reliably begin at a “cervical dilatation of 3 to 5 cm or more, in the presence of uterine contractions” [1]. However, investigators report that these criteria do not validly describe active labor onset for a large percentage of nulliparous women with spontaneous labor onset when traditional cervical dilation expectations are used to differentiate active from earlier labor [2,3]. The clinical dilemma is that many women are inadvertently admitted prior to progressive labor (i.e., pre-active labor) yet held to dilation rate expectations of active labor [4]. It is possible that women admitted early and given interventions aimed at accelerating labor progress (e.g., oxytocin augmentation) may be disadvantaged during labor in that such intervention may interrupt the time necessary for important physiological changes within the uterine and reproductive tissues to more fully manifest. This may, in part, explain why women admitted early in labor are more prone to oxytocin augmentation and are more than twice as likely to be delivered via cesarean [5-9].

Change in the activity of the enzyme lactate dehydrogenase (LDH) within uterine muscle during pregnancy and possibly throughout labor is a key physiological adaptation that may facilitate efficient uterine activity during labor. LDH is a predominantly intracellular, cytoplasmic enzyme that catalyzes the interconversion of pyruvate and lactate [Pyruvate+NADH+H^+^ ↔ (L)-lactate+NAD^+^], a process essential for adenosine triphosphate (ATP) production. LDH is composed of two different types of polypeptide chains, commonly called ‘H’ and ‘M,’ which combine to form either homotetramer isoenzymes composed of all ‘H’ chains [LDH_1_ (H_4_)] or all ‘M’ chains [LDH_5_ (M_4_)] or heterotetramer isoenzymes composed of a mixture of ‘H’ and ‘M’ chains [LDH_2_ (H_3_M_1_), LDH_3_ (H_2_M_2_), LDH_4_ (H_1_M_3_)]. The profile expression of LDH isoenzymes differs between body tissues depending on typical oxygen availability, e.g., more H-subunit dominant isoenzymes are available in tissues relying on aerobic metabolism, such as the heart, while M-subunit dominant isoenzymes are more abundant in tissues using anaerobic metabolism, such as skeletal muscle and liver [10-12]. The isoenzyme profile is also capable of adaptation *within* body tissues in response to appropriate signals, thus ensuring the tissue consistently maintains adequate ATP production.

The majority of studies measuring LDH levels during pregnancy are from the late 1950s through the 1970s. In myometrial muscle, LDH isoenzymes shift toward a more anaerobic profile as pregnancy advances, speculatively, to better equip the uterus to contend with hypoxic episodes related to labor contractions [10,13-15]. As a result, LDH_3_ and/or LDH_4_ have been reported to be in greatest concentrations in the pregnant myometrium at term [13,16,17]. Anaerobic shifting is important because otherwise intermittent hypoxia and resultant acidosis would rapidly reduce contractile force [18-21]. The pattern and timing of LDH isoenzyme profile shifting throughout late pregnancy and labor remains largely unknown.

LDH is released from its tissue of origin and enters the general circulation when cells are broken down or damaged. Because most tissues have LDH activities that are 500–700 times greater than that found in normal serum, a significant elevation of serum LDH occurs with even small amounts of tissue breakdown [22]. While total serum measurement of LDH provides only a non-specific measure of cellular breakdown/damage, determining specific LDH isoenzyme patterns is useful in the differential diagnosis of certain pathologic states. This is possible because tissue breakdown releases the isoenzymes contained within that particular tissue, leading to a change in the serum profile measured systemically. Isoenzyme measures can assist in diagnosing pathologic processes such as myocardial infarction, liver disease, and pre-eclampsia.

During normal labor, levels of total myometrial LDH decline from levels present before labor with a disproportionate decrease in ‘M’ dominant chains over ‘H’ dominant chains [14]. This finding aligns with reports that maternal serum total LDH concentrations are higher in the postpartum period than is normally found during the pre-labor period [23-28], peaking approximately 24–48 hours after delivery [23,29]. Given the tremendous amount of work performed and stress endured by the uterus during labor coupled with rapid uterine involution following delivery, it is likely that serum LDH isoenzyme levels measured after labor, in otherwise healthy women, predominantly reflect the enzymatic profile within the uterine muscle that existed during the labor period. This has been suggested by other research teams [14,23,24]. This means that LDH isoenzyme levels measured 24–30 hours after labor may serve as a retrospective indicator of uterine preparedness for labor.

The primary objective of this study was to describe relationships between maternal serum LDH isoenzymes measured 24–30 hours post-delivery and rates of cervical dilation during the first 4 hours following hospital admission for spontaneous labor onset. We also aimed to compare differences in post-delivery LDH profiles between women admitted to the hospital in pre-active versus established active labor. We hypothesized that better uterine preparedness for labor as evidenced by more efficient labor progression would be associated with a more anaerobic post-delivery serum LDH isoenzyme profile.

## Methods

A prospective study was conducted at a suburban, Midwestern hospital in the United States in which nearly 5000 women birth annually. Institutional Review Board (IRB) approval was granted (Mount Carmel IRB, study # 061130–3; The Ohio State University Biomedical IRB, protocol # 2006H0248) and written informed consents and Health Insurance Portability and Accountability Act authorizations were obtained from all women. Recruitment took place from 4/2007 – 2/2008 and was conducted by JN in the labor and delivery triage unit or in the labor room as soon after admission as possible. Approximately 70% of approached women accepted participation.

Participants were pregnant nulliparous women of low-obstetric risk (no significant medical history, absence of major pregnancy complications, e.g., pre-eclampsia or diabetes) admitted for spontaneous labor onset under criteria commonly associated with active labor onset, i.e., 3 cm to 5 cm cervical dilatation in the presence of regular uterine contractions (≥2 in a 10 minute window). Additional inclusion criteria were 18–39 years of age, 37–42 weeks gestation, singleton gestation, cephalic presentation, no identified fetal anomalies or growth issues, anticipated vaginal delivery, maternal weight <250 lbs, afebrile at study entry, and able to read and speak English. Augmentation of labor was permitted after labor admission although women undergoing labor inductions were not enrolled in the study. Care during labor was at the discretion of the labor care providers.

LDH total and isoenzyme concentrations were measured in maternal sera at two time points. The first sample was collected as near to the time of labor admission as possible with sampling occurring either concurrently with intravenous line placement, a standard facility order, or, less commonly, via a 20- or 22-gauge needle from the antecubital vein or below in the uncannulated arm. The second sample was collected 24–30 hours after vaginal delivery; typical patient discharge at this institution occurs 48 hours following vaginal birth. Each sample was collected into an 8.5 mL BD Vacutainer® serum separator tube (Reference # 367988) (Becton-Dickinson, Franklin Lakes, NJ). The blood was allowed to clot at room temperature for up 60 minutes and then centrifuged at 2500 rpm for 10 minutes. Sera were separated, immediately refrigerated, and determinations were performed within 48–72 hours after collection. Serum with visible hemolysis was discarded since the LDH released from the damaged erythrocytes might give spuriously high results. Hemolyzed samples were redrawn at the admission time point if labor had not progressed beyond the aforementioned labor onset criteria. At the post-delivery time point, hemolyzed samples were redrawn within 30 minutes of the initial sampling. For women who delivered via cesarean, post-delivery LDH samples were not collected due to the likelihood of these values being elevated secondary to surgical tissue damage. LDH measurements were made via a SYNCHRON LX System, an agarose gel electrophoresis system (Beckman Coulter, Inc., Fullerton, CA). Total LDH values were in units per liter (U/L) and the relative proportion of each LDH isoenzyme was expressed as a percentage (%). Absolute isoenzyme levels were not used because of the wide variability known to exist between individuals.

All digital cervical exams documented by labor care providers during the course of labor were transcribed *post hoc* from the labor record so that the average dilation slope for the first 4 hours post-admission could be determined. Since cervical exams are rarely performed at exactly 4 hours after the admission exam, slope calculations based on the exams immediately *prior to* and *after* the 4 hour time point were used to approximate dilatation at the 4 hour post-admission time point. The average dilation slope was then calculated for the first 4 hours post-admission; relationships between these rates (cm/hour) and the maternal serum LDH isoenzymes were then described for all subjects.

Finally, each subjects’ labor admission was retrospectively classified as either ‘pre-active labor’ or ‘established active labor’ based on the rate of cervical change during the first 4 hours after labor admission using *a priori* criteria. Between 3 cm and 5 cm dilatation, recent literature indicates that cervical dilation rates approximating >0.25 cm/hour for nulliparous women may, in some cases, indicate the potential onset of early active labor while rates ≥1 cm/hour are recognized to be indicative of established active labor [30-33]. Thus, for our study, a labor admission was classified as ‘pre-active’ when average dilation was ≤0.25 cm/hour for the first 4 hours post-admission labor or as ‘established active’ when average dilation was ≥1.0 cm/hour. Women having 4 hour post-admission dilation rates between >0.25 and <1.0 cm/hour were not included in comparisons because of the expected mix of women in pre-active and active labor in this range. Classifications of labor were determined before LDH results were available to the research team.

For data analyses, demographic variables were expressed as mean (SD) if continuous and as n (%) if categorical. Paired-analysis t tests with Bonferroni correction were used to test for differences between LDH measures at labor admission and 24–30 hours post-delivery. Spearman’s rho correlation coefficients were used to test for relationships between LDH measures and rates of cervical dilation during the first 4 hours following hospital admission because rates of dilation were not normally distributed; with a medium effect size (0.30), α = 0.05, and power = 0.80, a sample of 85 women achieving vaginal birth is required to test for correlations [34]. To test for LDH profile differences between the ‘pre-active’ and ‘established active’ labor groups, Student’s t tests were performed. We anticipated that this aim would be underpowered. P-values <0.05 were considered significant. Statistical analyses were made via SPSS Statistics 19 (IBM Corporation, Armonk, NY).

## Results

Ninety-one parturients were enrolled in the study and there was no attrition. Demographics of the sample are shown in Table 1. The majority of the sample self-classified as non-Hispanic whites. Among all women enrolled in the study, 81 birthed vaginally and 10 were delivered via cesarean. Among cesarean deliveries (n = 10), six were performed in the first-stage of labor (slow labor progression = 3; non-reassuring fetal heart patterns = 3) and four in the second-stage for arrest of fetal descent.

**Table 1:** Demographic variables (n = 91)

Maternal age (yrs)	24.9 (4.8)	Range: 18-36
Gestational age at delivery (days)	276.1 (7.1)	Range: 259-290
Hispanic		
Yes	5 (5.5%)	
No	86 (94.5%)	
Race		
White	66 (72.5%)	
Black	18 (19.8%)	
Other	7 (7.7%)	
Marital status		
Married	41 (45.1%)	
Not married	50 (54.9%)	
Body mass index (maternal)	29.9 (4.6)	Range: 18.0-41.7
Cervical dilatation at admission (cm)	3.6 (0.5)	Range: 3.0-5.0
Cervical effacement at admission		
50% – < 80%	11 (12.1%)	
≥ 80%	80 (87.9%)	
Mode of birth		
Vaginal	81 (89.0%)	
Cesarean	10 (11.0%)	
Membrane rupture type		
Spontaneous	33 (36.3%)	
Amniotomy within 4 hrs of admission	41 (45.0%)	
Amniotomy at > 4 hrs after admission	17 (18.7%)	
Oxytocin augmentation		
No	33 (36.3%)	
Yes, within 4 hrs of admission	27 (29.7%)	
Yes, at > 4 hrs after admission	31 (34.0%)	
Epidural use		
No	5 (5.5%)	
Yes	86 (94.5%)	
In-hospital labor duration (hr)*	8.9 (3.7)	Range: 2.8-20.9
Weight (infant) (g)	3392.8 (460.9)	Range: 2329-4722
Length (infant) (cm)	49.5 (2.2)	Range: 44.0-54.5

Mean (SD) for continuous variables; n (%) for categorical variables.

* Includes only those delivering vaginally (n = 81).

**Figure 1:**
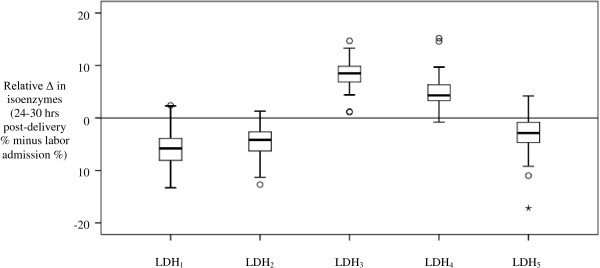
**Relative change in serum LDH isoenzyme paired-samples (n = 75).** p < 0.001 (2-tailed) for all isoenzyme changes.

LDH samples were collected from all subjects at labor admission although four specimens hemolyzed and repeat samples were not obtained; therefore, these specimens were not included in the analyses. At 24–30 hours post-delivery, LDH values were determined in 79 subjects; subjects delivered via cesarean were not sampled and two samples were unable to be collected due to early patient discharge. In all, serum paired-samples were obtained from 75 subjects and all differences were significant with Bonferroni correction (p < 0.001) (Table 2). Specifically, post-delivery total LDH, LDH_3_, and LDH_4_ had increased over values seen at labor admission while LDH_1_, LDH_2_, and LDH_5_ had decreased. Figure 1 displays relative LDH isoenzyme changes from admission to 24–30 hours post-delivery.

**Table 2:** **Maternal serum LDH paired-sample *t *tests between labor admission and post-delivery samples (n = 75)**

	**Total (U/L)**	**Isoenzyme (%)**				
		**1**	**2**	**3**	**4**	**5**
Labor admission	147.59 (22.81)	29.66 (3.13)	30.33 (3.17)	19.21 (2.36)	8.74 (2.18)	12.07 (3.88)
Post-delivery (24–30 hrs post)	173.35 (30.92)	23.89 (3.57)	26.00 (3.30)	27.45 (3.17)	13.62 (3.52)	9.05 (2.43)
*t* test value	7.491*	14.186*	14.055*	28.898*	14.851*	7.898*

Values are reported as mean (SD).

Each measure was normally distributed per the Kolmogorov-Smirnov test (p > 0.05). Bonferroni correction for multiple tests was p < 0.008 (i.e., p = 0.05/6).

*p < 0.001 (2-tailed).

Maternal serum LDH total and isoenzyme levels measured at labor admission held no correlation with rates of cervical dilation during the first 4 hours post-admission. However, serum LDH measures at 24–30 hours post-delivery yielded several significant relationships with post-admission cervical dilation rates. Specifically, as rates of cervical dilation during the 4 hour post-admission assessment period increased, percentage distributions of LDH_1_ decreased (Spearman’s rho = −0.246, p = 0.029) while LDH_3_ (Spearman’s rho = 0.267, p = 0.017) and LDH_4_ (Spearman’s rho = 0.268, p = 0.017) increased.

**Figure 2:**
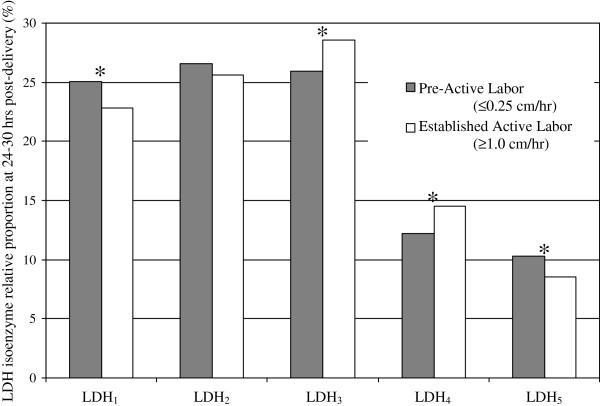
**Post-delivery serum LDH isoenzyme profile comparison between pre-active labor and established active labor admission groups.** *p < 0.05 (2-tailed).

Pre-active labor (≤0.25 cm/hour) (n = 22) and established active labor (≥1.0 cm/hour) (n = 23) groups did not demonstrate significant LDH differences at baseline. However, at 24–30 hours post-delivery, several LDH isoenzyme differences emerged between those admitted in pre-active versus established active labor (Figure 2). Specifically, women admitted in established active labor had higher relative serum levels of LDH_3_ (t = 2.373; p = 0.023) and LDH_4_ (t = 2.268; p = 0.029) and lower levels of LDH_1_ (t = 2.073; p = 0.045) and LDH_5_ (t = 2.041; p = 0.048) when compared to women admitted in pre-active labor. Forty-six women had 4 hour post-admission dilation rates between >0.25 and <1.0 cm/hour; thus, they did not qualify for either the pre-active or established active labor group. This group was not compared to the others due to the nebulous nature of their labor status.

Despite having similar cervical dilatations at admission (3.4 ± 0.5 and 3.7 ± 0.6 cm, respectively; NS), women admitted in pre-active labor had an in-hospital labor duration of 12.1 ± 4.3 hours while those admitted in established active labor had a duration of 5.3 ± 1.4 hours (p < 0.001). The difference in in-hospital labor duration between these groups resulted from differences in the time from admission until complete dilatation; second stage durations did not differ between groups. The oxytocin augmentation rate was 95.5% among women admitted in pre-active labor and 34.8% for those admitted in established active labor (χ2 = 18.064; p < 0.001). Interestingly, all three cesareans performed in the active phase for slow labor progression followed a pre-active labor admission.

Because skeletal muscle damage can contribute to serum LHD levels, it is noteworthy that parturients with perineal episiotomy/laceration ≥2 degree (n = 54) and those with <2 degree laceration or none (n = 25) did not differ on LDH measures at 24–30 hours post-delivery.

## Discussion

The significant correlations between rates of cervical dilation during the first four hours after labor admission and post-delivery LDH isoenzyme values support our hypothesis. Specifically, more efficient cervical dilation is associated with a more anaerobic LDH isoenzyme profile whereas slower labor progression is associated with a more aerobic profile. This also aligns with our finding that women admitted in progressive active labor had a more anaerobic post-delivery LDH profile as compared to women admitted in pre-active labor.

We postulate that post-delivery serum LDH profiles, sampled 24–30 hours after delivery, represent the enzymatic composition of uterine muscle surrounding the labor period since uterine muscle endures the greatest activity and stress during labor and maternal serum LDH levels peak at 24–48 hours after the tissue level event causing their release [23,29]. Others have reported that LDH anaerobic isoenzyme profile shifts occur in the human myometrium with advancing pregnancy [10,13-15] although it remains unknown when the shift is optimized. In non-pregnant states, estrogenic hormones are known to stimulate LDH activity in non-uterine tissue [35-38] with increased synthesis being predominantly of an anaerobic type [35,37,38]. In uterine muscle, positive correlations between estrogen and LDH levels have been demonstrated in non-pregnant states [39,40]. Since estrogens in maternal plasma and myometrium become more dominant with labor progression, terminating abruptly after delivery [1], we speculate that LDH shifting occurs throughout the labor continuum.

Our LDH-related findings indicate that women admitted in true active labor may have a distinct myometrial advantage in producing ATP during the hypoxic episodes that accompany uterine contractions, compared to those admitted in pre-active labor. Adverse consequences of oxytocin augmentation may be, at least partially, related to the fact that the uterine muscle isoenzyme environment may not yet be optimized for uterine contraction activity when this intervention is implemented. The significantly higher rate of oxytocin use among women admitted in pre-active labor is concerning because oxytocin, a “high-alert medication” [41], is the intervention most commonly associated with preventable adverse perinatal outcomes [42]. Receiving oxytocin at an earlier stage of labor is associated with a higher cesarean risk [9]. Incomplete LDH shifting may, in part, explain why women with spontaneous labor onset who are admitted earlier in labor (e.g. <4 vs. ≥4 cm) are more than twice as likely to be delivered via cesarean [5-9]. Rates of oxytocin supplementation and surgical birth in our small sample of women admitted in pre-active labor support these concerns.

This study was limited by a smaller than desired sample size especially in regards to testing for group differences. In addition, although uterine muscle is likely the key contributor to systemic maternal LDH measures in the peripartum period [14,23,24], other potentially contributing sources cannot be fully discounted. The placenta [17,39,43-45], skeletal muscle [14], and even intravascular hemolysis in the uterine sinusoids [23] have been suggested to be potential contributors to maternal measures. However, with the placenta being most represented by LDH_4_ and LDH_5_[16,46,47], skeletal muscle containing large concentrations of LDH_5_[14,22,48], and hemolysis leading the near-exclusive release of LDH_1_ and LDH_2_[22,48], one would expect that if any of these were key contributors to peripartum LDH increases, predominant increases in LDH_1_, LDH_2_, and LDH_5_ would have been found. Indeed, the relative measures of these three isoenzymes decreased in maternal serum between the admission and postpartum sampling time points (p < 0.001 for each). In the present study, relative and even absolute increases of LDH_3_ and LDH_4_ (p < 0.001) suggest that the uterine muscle is the most likely contributor to systemically measured LDH in the post-delivery period. These particular isoenzymes are the ones known to be in the greatest quantities in the myometrium of pregnant women [13,16,17]. Reproducing this study in a larger, more racially and/or ethnically diverse population would be a valuable scientific contribution.

## Conclusions

In this study, parturients progressing most slowly after being admitted for labor appear to have LDH isoenzyme profiles that are less equipped to contend with contraction-related anaerobic conditions compared to those parturients progressing most rapidly after admittance. Although serum LDH profile assessments cannot prospectively benefit clinicians in their admission decision-making, the findings presented in this study suggest that watchful patience rather than early intervention seems prudent when there is uncertainty regarding whether active labor has begun. Such patience may allow the time needed for important physiological changes within uterine muscle to more fully manifest.

## Competing interests

The authors declare that they have no competing interests.

## Authors’ contributions

JN designed the study, conducted the study protocol, performed the statistical analyses, and drafted the manuscript. NL assisted in interpreting findings and drafting the manuscript. EC participated in the design and coordination of the study and helped to draft the manuscript. All authors read and approved the final manuscript.

## Pre-publication history

The pre-publication history for this paper can be accessed here:

http://www.biomedcentral.com/1471-2393/13/128/prepub
